# PEG-ing down (and preventing?) the cause of pegloticase failure

**DOI:** 10.1186/ar4572

**Published:** 2014-05-30

**Authors:** Aryeh M Abeles

**Affiliations:** 1 Division of Rheumatology, University of Connecticut Health Center, 263 Farmington Avenue, Farmington, CT, 06030, USA

## Abstract

Pegloticase is a powerful but underutilized weapon in the rheumatologist’s armamentarium. The drug’s immunogenicity leads to neutralizing antibody formation and rapid loss of efficacy in roughly one-half of all patients, which remains an impediment to broader use. New data, however, suggest that drug survival might improve with concomitant immunosuppressive agent (s), which merits further study. Efficacy appears to be unchanged when pegloticase is infused at 3-week (rather than 2-week) intervals. Stretching the time between infusions may also improve patient adherence and allow for earlier identification of transient responders.

## 

In the previous issue of *Arthritis Research and Therapy*, Hershfield and colleagues published a study putting forth a number of novel and potentially important findings regarding pegloticase, a powerful but underutilized weapon in the small but growing anti-hyperuricemic arsenal [1].

The study examined the efficacy of pegloticase in a cohort of 30 patients with severe gout (93% tophaceous) utilizing an every 3-week infusion regimen, rather than the every 2-week schedule employed in previously published phase 3 trials. Despite the longer interval between infusions in the current study, the effectiveness of pegloticase is no worse (17/30 patients are persistent responders), and the pharmacokinetics of the drug suggest this should come as no surprise. The authors correctly note that a 3-week interval would be significantly more convenient for patients, and notably that such a regimen would also prove less costly to payors. Hershfield and colleagues’ dosing schedule would also help to identify transient responders earlier in the course of treatment – only four of 12 transient responders had uric acid >6 mg/dl 2 weeks after the first infusion (three of whom had previously been exposed to pegloticase in earlier phase 1 and 2 studies), whereas 11 of 12 transient responders had uric acid >6 mg/dl at 3 weeks (vs. only one of 17 persistent responders). For the reasons just elaborated upon, the paper demonstrates that dosing every 3 weeks may not just be as good as the current protocol, but may in some ways be superior.

Hershfield and colleagues also upend the assumption that neutralizing antibodies to pegloticase are formed against uricase itself. Their paper clearly demonstrates that neutralizing antibodies develop in response to the polyethylene glycol (PEG) moiety of the drug, a finding that rebuffs a recent commentary which suggested antibodies to PEG are not pathogenic [2]. A septe and larger study (169 patients exposed to pegloticase) published in the previous issue *Arthritis Research and Therapy* by Lipsky and colleagues reaches the same conclusion regarding anti-pegloticase antibodies: anti-PEG antibodies are responsible for loss of efficacy rather than antibodies to the uricase enzyme itself, the latter of which rarely occur (positive more than once in only 11 subjects) and occur much later during the course of treatment, long after neutralizing anti-pegloticase antibodies have developed [3]. This larger study also demonstrates that an anti-pegloticase antibody titer >1:2,430 generally predicts loss of efficacy to the drug.

Finally, and not least of all, Hershfield and colleagues’ smaller study included post-transplant patients (who were excluded from the phase 3 studies), a population particularly susceptible to developing gout. Of seven post-transplant subjects in the study, six proved to be persistent responders (86%) [1]. Although this is an admittedly small number of patients upon which to base any conclusion, it does raise the intriguing question of whether immunosuppression might lead to less of a mounted antibody response against pegloticase, and thus to more favorable outcomes. This is no small point; patients who are placed on pegloticase generally have severe, long-standing, and refractory gout, and should be given every chance to optimize their response to a potentially transformative therapy.

While this signal is worth pursuing, some questions are immediately raised: how immunosuppressed must patients be to prevent neutralizing anti-PEG antibody formation, and with what should this be accomplished? Of the small subcohort in this trial, all patients were on cyclosporine or mycophenolate mofetil, and five of seven patients were on a combination of immunosuppressive agents. In choosing an immunosuppressant for the express purpose of preventing neutralizing antibodies, the risk of these drugs would very probably outweigh any proposed benefit (cyclosporine in particular might be the least desirable immunosuppressant for a patient with severe gout, because it both increases serum uric acid levels and decreases the glomerular filtration rate).

If a trial was designed to investigate this line of query, a reasonable immunosuppressive agent of choice might be methotrexate. This drug has been shown to effectively prevent neutralizing antibodies from forming against monoclonal antibodies to anti-tumor necrosis factor [4,5]. Methotrexate’s inhibitory effect may not extend to preventing antibody formation against PEG, although methotrexate has also been shown to inhibit antibody formation against the polysaccharide 23-valent pneumococcal vaccine [6]. Methotrexate might also yield the unintended benefit of acting as an anti-inflammatory agent to suppress gouty attacks. However, because patients with severe gout have multiple comorbidities that place them at higher risk for medication side effects, methotrexate should not be considered in the clinical setting in the absence of data to support its use [7]. Nevertheless, methotrexate therapy is an avenue of inquiry that is clinically relevant and needs exploration to increase the likelihood that patients who begin this powerful drug can remain on it.

## Abbreviations

PEG: Polyethylene glycol.

## Competing interests

The author declares that he has no competing interests.
